# Multifactorial Modulation of Food-Induced Anaphylaxis

**DOI:** 10.3389/fimmu.2017.00552

**Published:** 2017-05-16

**Authors:** Sara Benedé, María Garrido-Arandia, Laura Martín-Pedraza, Cristina Bueno, Araceli Díaz-Perales, Mayte Villalba

**Affiliations:** ^1^Dpto. Bioquímica y Biología Molecular I, Universidad Complutense de Madrid, Madrid, Spain; ^2^Centro de Biotecnología y Genómica de Plantas (UPM-INIA), Campus de Montegancedo, Pozuelo de Alarcón, Madrid, Spain

**Keywords:** food-induced anaphylaxis, IgE, allergens, diet, mast cells, basophils

## Abstract

Prevalence of food-induced anaphylaxis increases progressively and occurs in an unpredictable manner, seriously affecting the quality of life of patients. Intrinsic factors including age, physiological, and genetic features of the patient as well as extrinsic factors such as the intake of drugs and exposure to environmental agents modulate this disorder. It has been proven that diseases, such as mastocytosis, defects in HLA, or filaggrin genes, increase the risk of severe allergic episodes. Certain allergen families such as storage proteins, lipid transfer proteins, or parvalbumins have also been linked to anaphylaxis. Environmental factors such as inhaled allergens or sensitization through the skin can exacerbate or trigger acute anaphylaxis. Moreover, the effect of dietary habits such as the early introduction of certain foods in the diet, and the advantage of the breastfeeding remain as yet unresolved. Interaction of allergens with the intestinal cell barrier together with a set of effector cells represents the primary pathways of food-induced anaphylaxis. After an antigen cross-links the IgEs on the membrane of effector cells, a complex intracellular signaling cascade is initiated, which leads cells to release preformed mediators stored in their granules that are responsible for the acute symptoms of anaphylaxis. Afterward, they can also rapidly synthesize lipid compounds such as prostaglandins or leukotrienes. Cytokines or chemokines are also released, leading to the recruitment and activation of immune cells in the inflammatory microenvironment. Multiple factors that affect food-induced anaphylaxis are discussed in this review, paying special attention to dietary habits and environmental and genetic conditions.

## Introduction

The widely used definition of anaphylaxis as “an adverse allergic reaction that is rapid in onset and may cause death” is also accompanied by clinical criteria for diagnosis (1). Other definitions of anaphylaxis have been formulated to aid its diagnosis and management (2). It constitutes an alarming medical emergency (1, 3, 4), not only for the patient and members of the family, but sometimes also for the healthcare professionals involved. Death usually occurs because of respiratory or cardiac arrest as an aftershock of an anaphylactic attack (5). Although life-threatening episodes are uncommon, these events constitute an unpredictable risk and their prevalence is steadily increasing affecting up to 2% of the population (6). Hospital and critical care unit admissions are not common but continue to increase, doubling in frequency between 1998 and 2012 (7, 8). An accurate population-based estimate is difficult to obtain due to underdiagnosis and underreporting, as well as by the use of different clinical definitions for anaphylaxis and methods of case diagnosis in populations under study (9).

Molecules such as histamine (HIS), tryptase, leukotrienes, and prostaglandins, among others, mediate the clinical manifestations of anaphylaxis. Secretion of these mediators occurs after an allergen cross-links the IgEs bound to mast cells (MCs) and basophils. However, IgE-independent immune mechanisms may also be involved (3). Physiological state (3), as well as certain diseases and medications (10), are risk factors for anaphylaxis. Cofactors such as drugs or exercise that can exacerbate or trigger acute anaphylactic episodes have been described (11–13). Specialist physicians and patients need to be aware of the relevant risk factors and cofactors in the context of long-term management and treatment of this condition. In this mini review, we summarize the physiological, genetic, and environmental aspects in the field of food allergy (FA) focusing special attention on anaphylactic reactions.

## Incidence of Food-Derived Anaphylaxis

Food allergy is a serious and often life-threatening health concern that is increasing in frequency especially in the vulnerable pediatric population affecting 4% of children and 2–3% of the adult population worldwide (14). The treatment requires changes in dietary habits and social behavior (15). FA is originated by a reaction of the immune system that results in non-tolerance of specific foods. In most patients, IgE mediates this immune disorder, although there are also IgE-independent cell-mediated allergies that are accompanied by gastrointestinal symptoms (16), but they are not going to be analyzed in this review.

More than 170 foods have been associated with type I allergies, the most common of which are milk, egg, wheat, fish, shellfish, peanuts, soy, and tree nuts, although the prevalence varies geographically (17). The two most frequent food allergens that induce severe and potentially lethal anaphylaxis are milk and egg, while the third differs between countries (18) being peanuts in the USA and Switzerland, wheat in Germany and Japan, tree nuts in Spain, and sesame in Israel (16). Foods and meals containing hidden allergens at restaurants are a serious source of risk for patients with food allergies (19). The prevalence of food-induced anaphylaxis seems to vary with the population selected and the region where they are recruited. The point prevalence of food challenge-confirmed allergy is under 1% (20–23).

Food allergies can be caused by primary sensitization to an eliciting food allergen (class I allergen), or they can be triggering by a primary sensitization to an inhalant allergen and later IgE cross-reactivity to a homologous protein in food (class II allergen) (24). Almost all plant food allergens are either storage or defense-related proteins and three dominating plant allergenic protein superfamilies have been identified as being involved in triggering severe reactions. These are prolamins that include the 2S albumins, lipid transfer proteins (LTPs), and α-amylase/trypsin inhibitors, cupins that comprise the major globulin storage proteins, mainly found in legumes and nuts and the Bet v 1-related protein family (25, 26). Among animal food allergen families, parvalbumins, tropomyosins, and caseins are the three dominant groups (25, 26). The most significant molecular characteristic of these allergens is their resistance to proteolysis, which increases the probability of reaching intact the intestinal mucosa and triggering an immune response (27).

## Influence of Diet in FA

**Figure 1:**
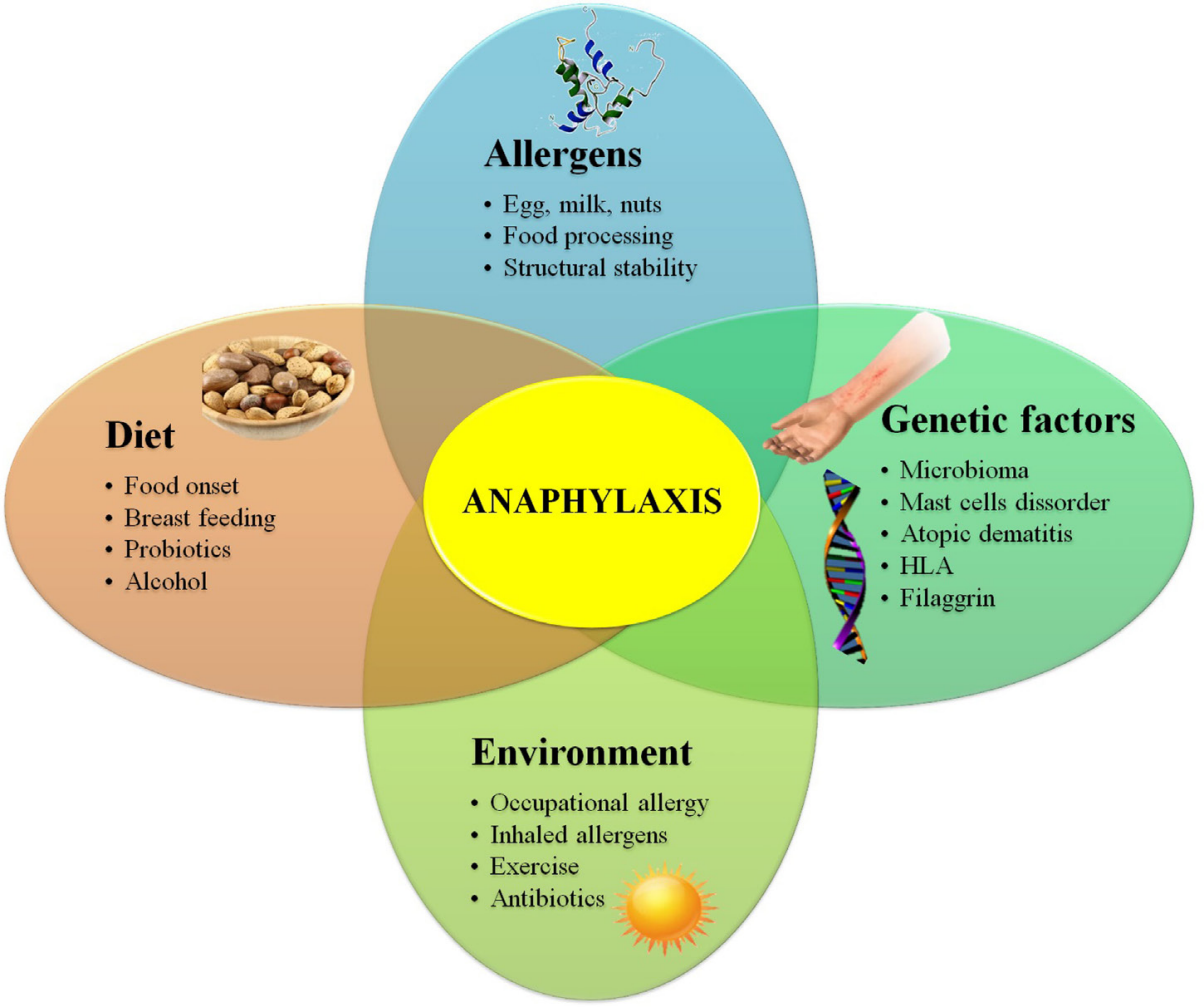
**Diagram showing genetic, environmental, dietary habits, and allergen-related risk factors for food allergy**.

The prevalence of FA is growing at an alarming rate (28), and it is not easy to know with precision the reasons behind this growth. Factors such as a sterile pathogen-free environment, changing dietary habits, vitamin D deficiency, intestinal microbiota composition, stability properties of certain allergens, or alcohol consumption may play a role in this increase (29) (Figure 1).

Breast milk provides an abundant source of soluble IgA and prebiotic glycans that promote the expansion of species of *Lachnospira, Veillonella*, and *Rothia*, which are adapted to this food source (30, 31). In contrast, formula feeding may not be sufficient for neonates with poor immunity, exacerbating the allergic reactions and only amino acid-based formulas should be considered as non-allergenic. Pre- or probiotic supplemented infant formulas are also used but they might not fully replicate the beneficial effect of breast milk.

The correct time to incorporate certain allergenic foods in the diet of infants has also been controversial topic. It has been demonstrated that the early introduction of peanut to high-risk 7-month-old babies reduces the incidence of peanut allergy (32). Moreover, the LEAP study showed that in high-risk infants, sustained consumption of peanut beginning in the first 11 months of life was highly effective in preventing the development of peanut allergy (33).

Nowadays, food processing is an important aspect in our daily diet. The stability of the allergenic proteins during these procedures (34) and the resistance to gastric and intestinal digestion (35) are properties that preserve the integrity of IgE-specific epitopes, as occurs in LTPs allergic patients (36). These processes can alter structural characteristics of allergens and therefore modify their allergenic capacity (37–40). Patients, who possess specific IgE to linear epitopes, have a stronger response to cooked and partially digested antigens, while those patients who recognize conformational or three-dimensional epitopes have a milder clinical response (41, 42).

It has also been proved that some nutrients, such as vitamin D, influence the regulation of the immune system (43–45). In fact, the frequency of food anaphylaxis increases in areas where the exposure to UVB radiation is low and vitamin D is not synthesized in adequate concentrations (46). Moreover, vitamin D deficiency is associated with challenge-proven FA in infants (47). The deficit of this vitamin promotes Th2 responses by reducing the number of Th1 cells and inducing Th2 cell proliferation. However, in correct doses, vitamin D is responsible for reducing the allergic response by promoting Treg and suppressing Th17 cells, demonstrating the importance of a correct balance (48).

## Genetic and Environmental Factors Involved in FA

A few specific gene mutations have been related to food anaphylaxis. Several studies have identified an association of HLA genes with peanut allergy (49–51). Moreover, filaggrin is a key protein in the function of the epidermic epithelial barrier, and Cabanillas and Novak (52) have shown that the development of peanut allergy in children that carry one or more mutations in the filaggrin genes is provoked even when they are exposed to very small quantities of allergen, a risk that increased with higher dose exposure.

However, exclusively genetic susceptibility cannot explain the rapidly increasing prevalence of food allergies, suggesting that something in our environment is promoting this disease. There is increasing evidence that an early sensitization is occurring in individuals through the skin or even through breast milk and the amniotic fluid. Atopic dermatitis, multiplies by a factor of 10 the risk of suffering peanut allergy (53). The exposure to peanut particles suspended in the environment (54), the absorption of active allergenic components present in topical lotions (55), the damage induced by scratching, or the presence of Staphylococcal enterotoxin B in the areas surrounding a skin lesion have all been associated to peanut allergy (56). Moreover, workers exposed to occupational food allergens can be sensitized through the skin or by inhalation (57, 58).

Allergic reactions are increasingly associated with demographic variables, which are becoming every day more widespread in the twenty-first century such as cesarean births. Delivery by cesarean section may predispose the newborn baby to FA, presumably due to modifications in the establishment of gut microbiota caused by a different initial exposure to microbes (59, 60). Continuous intake of antibiotics has a profound influence on the bacterial composition that colonize the intestinal track, which may affect the allergic response to food and which seems to be essential for the maintenance of homeostasis (61, 62). In a healthy gastrointestinal tract, the epithelium develops immune tolerance to antigens present in the diet, fights invading microorganisms, and limits their presence near the mucosa (63, 64). Alterations in gut microbiota (dysbiosis) and consequent disruption of homeostasis have been linked to the occurrence of allergic reactions (65). However, whether an incremented permeability in the intestine is a cause or a consequence of an allergic reaction is still an open question (66). The finding that Clostridia strains can suppress allergy in mice (67, 68) suggests the potential use of microbial therapies to enhance the development of tolerance when given with allergen immunotherapy (69, 70).

A broad variety of other factors may also contribute to the increased risk of food-induced anaphylaxis. A prospective study reported an association between anaphylaxis and exercise, drug use, acute infection, premenstrual status, or psychological stress in 20% of patients (13). Food-dependent exercise-induced anaphylaxis has also been observed, although it is found more often in adults than in children (71–73). Studies have also shown that epigenetic variants, primarily in the pattern of DNA methylation, are associated with FA (74). Increased severity of anaphylaxis has also been reported for the elderly, patients with pre-existing cardiovascular disease, MC disorders, or undergoing concomitant treatment with a beta-adrenergic blocker and/or angiotensin converting enzyme inhibitor (75). Non-steroidal anti-inflammatory drugs also seem to enhance some food-allergic reactions (76).

## Mucosal Immunology of FA

**Figure 2:**
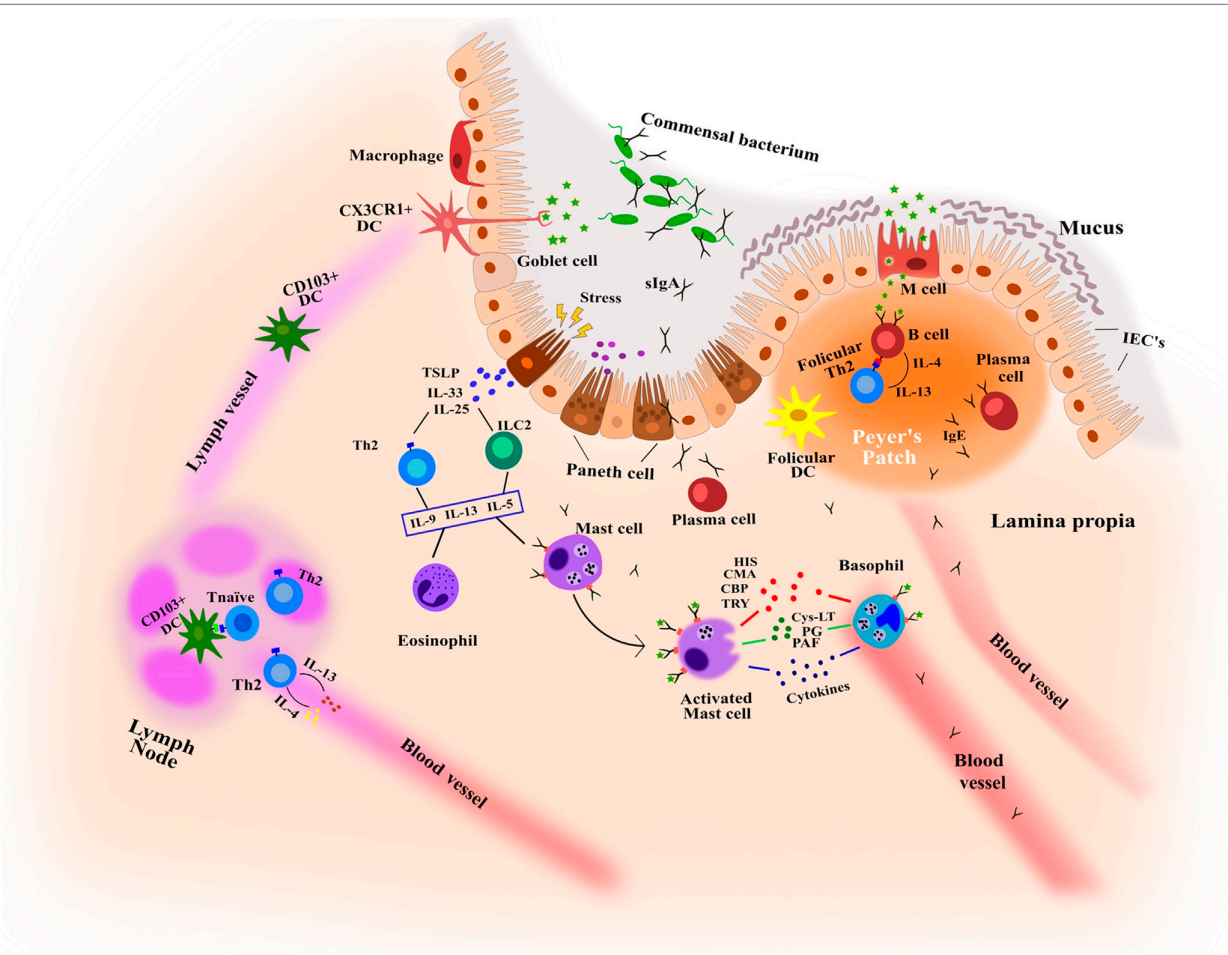
**Key features in the immunological mechanisms of intestinal mucosa involved in food allergy and food-induced anaphylaxis**. CBP, carboxipeptidase; CMA, chymase; Cys-LT, cysteinyl leukotriene; HIS, histamine; IECs, intestinal epithelial cells; PAF, platelet-activating factor; PG, prostaglandins; TRY, tryptase.

The gastrointestinal tract has a large surface area that is responsible for the digestion and absorption of food. It is formed by the intestinal epithelium, a cell monolayer that creates a selective barrier separating both outside and inside environments (77) (Figure 2). This epithelium is the first line of defense forming a physical and biochemical barrier where the mutualistic relationship between commensal microbial communities and immune cells are in a state of homeostasis (78), thereby preventing the colonization by other pathogenic microorganisms (79). This delicate balance depends on the functions of intestinal epithelial cells (IECs), maintaining a state of non-responsiveness (tolerance) or releasing an antipathogenic immunity.

Enterocytes form the dominant cell population in the epithelial barrier, constituting more than 80% of all IECs with functions that are metabolic and digestive, as well as secretory. Globet and paneth cells, also present in the barrier, release numerous hormone regulators such as mucins and antimicrobial proteins and protect the host from infection (80, 81). The most abundant mucins, MUC2, control macrophage and adaptive T cell responses during inflammation and inhibit pathogen microorganism chemotaxis (82, 83). Microfold cells (M cells) that form concentrations in the epithelium above Peyer’s patches mediate the presentation of antigens and microorganisms to the mucosal immune system, appearing as efficient mechanisms of receptor-mediated transport (84). In addition, among the cells of the immune system, dendritic cells and macrophages play an important role as antigen presenting cells. They are able to capture, process, and present the antigen to T lymphocytes, and induce a specific immune response. On the other hand, MCs located in the lamina propria and cell barrier detect antigens through the specific IgEs attached to their membrane and induce inflammatory reactions by secreting cytokines and immune mediators.

## Cells and Molecules Involved in Food-Induced Anaphylaxis

### Effector Cells of Anaphylaxis

Mast cells and basophils represent the primary effector cells in the pathophysiological process of food-induced anaphylaxis (85, 86), although it has been speculated that they may also participate in late-phase and chronic allergic reactions (87). They express complementary and overlapping roles in both regulatory and effector activities (88) and due to the similarities in histochemical characteristics, IgE receptor expression and the mediators produced, MCs and basophils were supposed to be related. However, transcriptional analysis has shown minimal similarity between MCs and basophils (89), and in recent years, several studies have indicated that although derived from unique hematopoietic progenitors, they are not closely related (90, 91). Both cell types also differ in their development (92). Basophils complete their differentiation within the bone marrow, while MCs circulate in the blood as progenitor cells and enter the tissues to proliferate and maturate (93). Tissue microenvironment regulates the mast cell expression of proteases (94, 95). Accordingly, humans MCs are classified based on their protease composition as MCs containing both tryptase and chymase, typically present in the bowel submucosa within the connective tissue, and those containing only tryptase, which are predominant on mucosal and epithelial surfaces such as bowel mucosa (96). Chymase-only-positive MCs have also been described but they appear to be very infrequent (97).

### Mechanism of Effector Cell Signaling

To understand the mechanisms of effector cell activation, it is essential to comprehend the regulation of intracellular signaling pathways that lead cells to release their mediators during the acute phase of anaphylaxis. In the last years, these events have been extensively studied and reviewed in the literature (98–104). However, given the complexity of these processes, here we present only a brief overview of the current state of affairs, focusing primarily on FcεRI. Signaling initiated by FcεRI aggregation triggers phosphorylation of immunoglobulin receptor activation motifs, which is mediated by the Src family tyrosine kinase Lyn and Syk. Subsequently, two major signaling enzymes, phosphoinositide phospholipase C-gamma and phosphoinositide 3-kinase, are activated which causes the release of calcium from intracellular stores, leading to cell degranulation, arachidonic acid metabolism, and activation of transcription factors with the consequent production and release of lipid mediators and the production of cytokine and chemokine. Effector cells also possess receptors characterized by a conserved immunoreceptor tyrosine-based inhibitory motif that when phosphorylated, recruits protein (SHP-1) and lipid phosphatases (SHIP1, SHIP2) that inhibit protein interactions and therefore inhibit the signaling cascade. In the end, despite all these complex cellular signaling pathways, the status of effector cell activation is really a balance between the pathways that upregulate these processes and those that downregulate them.

### Mediators of Anaphylaxis

Both MCs and basophils participate in clinical manifestations of anaphylaxis that typically involve the skin and the respiratory and gastrointestinal tracts (105). They release potent inflammatory mediators after an antigen cross-links the specific IgE attached to their surface receptors. Tissue-derived MCs and circulating basophils can immediately release preformed mediators stored in their granules such as HIS, heparin, or proteases that are responsible for many of the acute symptoms such as vascular leak or bronchoconstriction (106, 107). HIS, the main biogenic amine released upon activation of effector cells, has long been proven to be a short half-life factor triggering a variety of symptoms of anaphylaxis, including inflammation, itchiness, and mucus production (108). Recent studies have shown a correlation between plasma HIS levels and anaphylaxis severity (109). The efficiency of blocking HIS H1 and H4 receptors to suppress intestinal anaphylaxis in peanut allergy has also been proved, being this effect mediated through the limitation of mesenteric lymph node and intestinal dendritic cell accumulation and function (110). Tryptase is currently one of the biomarkers to assess MCs activation and levels are enhanced after onset of anaphylaxis (111). Basal tryptase levels in serum may predict moderate to severe anaphylaxis in children with FA (112) and they have also been described as good markers for the diagnosis of food-induced anaphylaxis (113). Chymase and carboxypeptidase levels in serum of patients with anaphylaxis have been found to be significantly greater than those found in healthy subjects, although there is no correlation with tryptase levels (114).

After activation, MCs and basophils can also rapidly synthesize lipid compounds in their membrane. These include prostaglandins, leukotrienes, and platelet-activating factor (PAF) that are mediators of hypotension, bronchospasm, mucus secretion, as well as leukocyte and dendritic cell recruitment during anaphylaxis (115). Increased urinary concentrations of leukotrienes and prostaglandins have been found in patients with food-induced anaphylaxis (caused by seafood, nuts, and soybean milk) compared to healthy individuals, showing good correlation between both mediators (116). PAF correlates better than either HIS or tryptase with severity of symptoms (117), and a deficiency of PAF-AH, the enzyme that inactivates PAF, predisposes patients to severe anaphylaxis (118). Moreover, it has been proven that detection of malfunction of PAF-AH may help identify individuals at risk of anaphylaxis (119).

Upon activation of MCs and basophils, cytokines or chemokines are newly synthesized and released, which are involved in the recruitment and activation of several cells in the inflammatory microenvironment (106). Many cytokines, chemokines, and growth factors are released from MCs (120) and basophils (121). IL-2, IL-4, IL-5, IL-6, IL-10, IL-13, and IFN-γ are elevated in anaphylactic patients (106), but only IL-2, IL-6, and IL-10 have been correlated with initial reaction severity (106, 109), although it is difficult to determine their origin because they are not exclusively produced by effector cells.

## Conclusion

Despite efforts to prevent FA and anaphylaxis and to elucidate the fundamental underlying mechanisms that link allergen exposure to symptoms, the incidence is increasing in developed countries. Individual genetic predisposition is an important determinant, although the features of the allergen itself and a variety of environmental factors, including diet and intestinal microbiota may have an important impact on sensitization, severity, and persistence of FA. Preventive strategies, such as dietary interventions, the use of probiotics, or prolonged exclusive breast-feeding, among others, have recently come into question and therefore it is important to identify and study how to control the risk factors to reduce the development of FA. The complex network of factors involved in these disorders makes research in this field difficult and much more investigation needs to be done to identify and manipulate the modifiable risk factors to offer significant benefits to mitigate this disease in the future.

## Author Contributions

SB, MG-A, LM-P, AD-P, and MV contributed to writing and critically revised the paper. CB contributed to designing and drawing the figures.

## Conflict of Interest Statement

The authors declare that the research was conducted in the absence of any commercial or financial relationships that could be construed as a potential conflict of interest.

## Abbreviations

FA, food allergy; HIS, histamine; IECs, intestinal epithelial cells; LTP, lipid transfer protein; MCs, mast cells; PAF, platelet-activating factor.
